# Supplementary material for: Patterns of Intron Gain and Loss in Fungi
Source: PLoS Biol. 2004 Nov 30;2(12):e422. doi: 10.1371/journal.pbio.0020422 (PMC532390; doi:10.1371/journal.pbio.0020422)
Supplement: Table S1 — Also available at http://genes.mit.edu/NielsenEtAl/. (4.3 MB ZIP). [file pbio.0020422.st001.zip › NielsenEtAl/html/258.html]

AN0733.1.NCU01635.1.MG01159.1.FG04290.1


```
 CLUSTAL W (1.82) Multiple Sequence Alignments - Introns Inserted


Sequence 1: NCU01635.1	136 aa
Sequence 2: FG04290.1	144 aa
Sequence 3: MG01159.1	168 aa
Sequence 4: AN0733.1	141 aa
Alignment Length: 181 aa
Number Identitical Residues: 133 aa
Alignment Score (without introns) 4632


MG01159.1 	MARTKQTAR-----~KSTGGKAPRKQLASKAA~--------RKSAPSTGGVKKPHRYKPG
NCU01635.1	MARTKQTAR-----~KSTGGKAPRKQLASKAA~--------RKSAPSTGGVKKPHRYKPG
FG04290.1 	MARTKQTAR-----~KSTGGKAPRKQLASKAA1SSLTSSSARKSAPSTGGVKKPHRYKPG
AN0733.1  	MARTKQTARKSSLC1KSTGGKAPRKQLASKAA~--------RKAAPSTGGVKKPHRYKPG
          	*********.:: . *****************         **:****************

MG01159.1 	~TVALREIRRYQKSTELLIRKLPFQRL~VSFSDRSSEHSANRRSITVEAVANIVCAQVRE
NCU01635.1	~TVALREIRRYQKSTELLIRKLPFQRL0V-----------------------------RE
FG04290.1 	~TVALREIRRYQKSTELLIRKLPFQRL0V-----------------------------RE
AN0733.1  	1TVALREIRRYQKSTELLIRKLPFQRL~V-----------------------------RE
          	 ************************** *                             **

MG01159.1 	IAQDFKSDLRFQSSAIGALQESVESYLVSLFEDTNLCAIHAKRVTIQS0SLQKDIQLARR
NCU01635.1	IAQDFKSDLRFQSSAIGALQESVESYLVSLFEDTNLCAIHAKRVTIQS~---KDIQLARR
FG04290.1 	IAQDFKSDLRFQSSAIGALQESVESYLVSLFEDTNLCAIHAKRVTIQS~---KDIQLARR
AN0733.1  	IAQDFKSDLRFQSSAIGALQESVEAYLVSLFEDTNLCAIHAKRVTIQS~---KDIQLARR
          	************************:***********************    ********

MG01159.1 	LRGERN
NCU01635.1	LRGERN
FG04290.1 	LRGERN
AN0733.1  	LRGERS
          	*****.
```
